# Prognostic and clinicopathological value of Slug protein expression in breast cancer: a systematic review and meta-analysis

**DOI:** 10.1186/s12957-022-02825-6

**Published:** 2022-11-14

**Authors:** Zhihao Zhang, Tian Fang, Yonggang Lv

**Affiliations:** 1 Department of Thyroid Breast Surgery, Xi’an NO.3 Hospital, the Affiliated Hospital of Northwest University, Xi’an, Shaanxi 710018 People’s Republic of China; 2 Department of Medical Oncology, Cancer Center, West China Hospital, Sichuan University, Chengdu, People’s Republic of China

**Keywords:** Meta-analysis, Breast cancer, Slug, Protein, Prognosis

## Abstract

**Background:**

Many studies have reported the relationship between prognosis and Slug protein expression in breast cancer patients, but the results are discrepant. Therefore, there is a need for meta-analyses with high statistical power to investigate and further explore their relationship.

**Methods:**

We used PubMed, Embase, the Cochrane Library, Scopus, MEDLINE, and the Web of Science to find studies on breast cancer and Slug. Overall survival (OS) and disease-free survival (DFS) were the study’s primary endpoints. We pooled hazard ratios (HRs) and odds ratios (ORs) to assess the association between Slug protein expression and prognostic and clinicopathological parameters. This study was performed using STATA version 14.0 for data analysis. (Stata Corporation, TX, USA).

**Results:**

We conducted a literature search by searching six online databases. Ultimately, we obtained eight studies including 1458 patients through strict exclusion criteria. The results showed that increased Slug protein expression resulted in poorer OS (HR = 2.21; 95% CI = 1.47–3.33; *P* < 0.001) and DFS (HR = 2.03; 95% CI = 1.26–3.28; *P* = 0.004) in breast cancer patients. In addition, the results suggested that breast cancer patients with increased Slug protein expression had a higher TNM stage (I–II vs III–IV; OR = 0.42; 95% CI = 0.25–0.70; *P* = 0.001), a greater tendency to have axillary lymph node metastases (N+ vs N0; OR = 2.16; 95% CI = 1.31–3.56; *P* = 0.003) and were more prone to estrogen receptor deficiency (positive vs negative; OR = 0.67; 95% CI = 0.45–0.99; *P* = 0.042). However, Slug protein expression was not associated with age, histological grade, tumor size, progesterone receptor status, or human epidermal growth factor receptor 2 status in breast cancer patients.

**Conclusion:**

This meta-analysis showed that elevated Slug protein expression may be related to poor outcomes in patients with breast cancer. Therefore, Slug is not only an indicator of patient survival but may also become a new target for breast cancer therapy.

**Supplementary Information:**

The online version contains supplementary material available at 10.1186/s12957-022-02825-6.

## Introduction

Breast cancer is the most common malignancy among women worldwide and is one of the leading causes of death among women aged 20–50 years [1]. In the 2020 Global Cancer Statistics, the number of breast cancer cases overtook lung cancer as the world’s most prevalent malignancy [2]. The annual incidence rate is still on the rise [3]. Breast cancer survival rates improve as systemic treatment strategies become more abundant [4]. However, the lack of effective predictors of disease progression and the widespread drug resistance in breast cancer means that these therapies remain unsatisfactory for some patients with breast cancer [5]. Therefore, it is imperative that researchers identify precise biomarkers of breast cancer and potential therapeutic targets for the treatment of the disease to improve survival [6, 7].

Snail family zinc finger 2 (Slug) is a C_2_H_2_ zinc-finger transcriptional repressor belonging to the three-member family of snail proteins (Snail, Slug, and Smuc), which mediates sequence-specific interactions with DNA [8], and has many biological functions, such as cell migration, cell invasion, cell cycle regulation, and stem cell characteristics in tumor cells [9]. In addition, reports have suggested that Slug protein expression is increased in various cancer cells, including lung, breast, ovarian, pancreatic, and colorectal cancers [10]. Previous studies have demonstrated that Slug affects breast cancer progression at many stages [11–13]. For example, slug acts as an important signaling pathway that promotes the proliferation and migration of breast cancer cells [14, 15]. Moreover, Slug induces and maintains the tumorigenic capacity of breast cancer cells [16]. Several studies have found that elevated Slug protein expression in breast cancer cells may be associated with multiple drug resistance, including resistance to chemotherapy and endocrine therapy [17, 18].

Many studies have explored the role of Slug protein in the clinicopathological parameters and prognosis of breast cancer, but the results were inconsistent [19–21]. Thus, it is essential to conduct a meta-analysis with high statistical power to study the role of Slug protein in the progression of breast cancer.

## Methods

The meta-analysis, as a traditional research method, provides convincing and reliable evidence related to medical health. Their value is particularly evident when their studies show similar clinically essential effects [22]. A detailed description of the meta-analysis can be found in the Additional file 1: Supplementary file. This study was registered with the International Prospective Register of Systematic Reviews database (PROSPERO), and the identification code is CRD42021224716. All aspects of the Preferred Items for Reporting of Systematic Reviews and Meta-Analyses (PRISMA) were followed [23].

### Search strategy

We conducted this study by searching PubMed, Embase, Cochrane Library, Scopus, MEDLINE, and Web of Science (for dates up to June 26, 2022) with the keywords: “SLUG” or “SNAIL2” or “SNAI2”, “breast cancer” or “breast neoplasm” or “breast tumor”. For example, the search query in PubMed was (((Breast Neoplasms [MeSH Terms]) OR (breast cancer)) OR (breast tumor)) AND (((SNAI2[MeSH Terms]) OR (SLUG)) OR (SNAIL2)). In addition, a further manual search of the reference lists of eligible studies was conducted to identify additional relevant studies.

### Inclusion and exclusion criteria

The inclusion criteria of this meta-analysis were as follows: (1) all studies had prognostic or clinicopathological outcomes. (2) Slug protein expression was analyzed in all breast cancer patients; (3) Hazard ratios (HRs) with confidence intervals (CI) could be extracted directly or inferred from Kaplan-Meier curves. (4) Clinical parameters for calculating the odds ratio (OR) can be extracted. The exclusion criteria: (1) conference abstracts, letters, reviews, meta-analyses, and animal model studies were excluded. (2) Studies that did not provide sufficient data were excluded.

### Data extraction and assessment of quality

Two researchers (Zhang and Fang) extracted the data for the studies separately. When disagreements arose, they were resolved through discussion. Data extraction included author, country, year of publication, number of patients, mean age (years) and median follow-up time (months), survival data, and clinicopathological parameters. In addition, Slug antibody dilution, location, and critical values were extracted. HRs for assessing prognosis were extracted directly from the paper, or if HRs could not be extracted directly, we chose to estimate HR from Kaplan-Meier curves via Engauge Digitizer Version4.1 (http://markummitchell.github.io/engauge-digitizer/). The quality of articles was evaluated using the Newcastle Ottawa Scale (Nos) [24], which has a maximum score of 9, and we discarded articles with a score of 5 or less.

### Data synthesis and statistical analysis

HR was used as an effect size indicator to assess the relationship between Slug protein expression and patient survival. An HR > 1 indicates that increased Slug expression is detrimental to the survival of breast cancer patients. For studies where HR was not provided, we extracted data from Kaplan-Meier curves using Engauge Digitizer Soft 4.1 (http://markummitchell.github.io/engauge-digitizer/) [25]. Using log-rank tests, we then replicated the Kaplan-Meier curves (GraphPad Software) and estimated HRs and their 95% CIs.

The OR represents the relationship between Slug expression and clinical parameters. We extracted clinical parameters for which we could calculate the OR and its CI and imported the data into STATA, where the OR values could be generated automatically. OR > 1 represents an increase in slug expression associated with more severe clinical parameters.

STATA version 14.0 (Stata Corporation, TX, USA) software was used for data aggregation and analysis. Effect size assessment is expressed as a pooled HR or OR with 95% CI. Statistical significance is expressed as a pooled *P*, and a *P* < 0.05 was considered statistically significant. The Cochran *Q* test and *I*^2^ statistics were used to measure heterogeneity; *P* < 0.1 and *I*^2^ value > 50% represented substantial heterogeneity [22]. At this point, the random model was used, and subgroup analysis was performed to detect potential heterogeneity. Otherwise, the fixed-effects model was used [26]. Sensitivity analysis was used to check data stability, and Egger’s test was used to detect publication bias.

## Results

### Search results

**Fig. 1:**
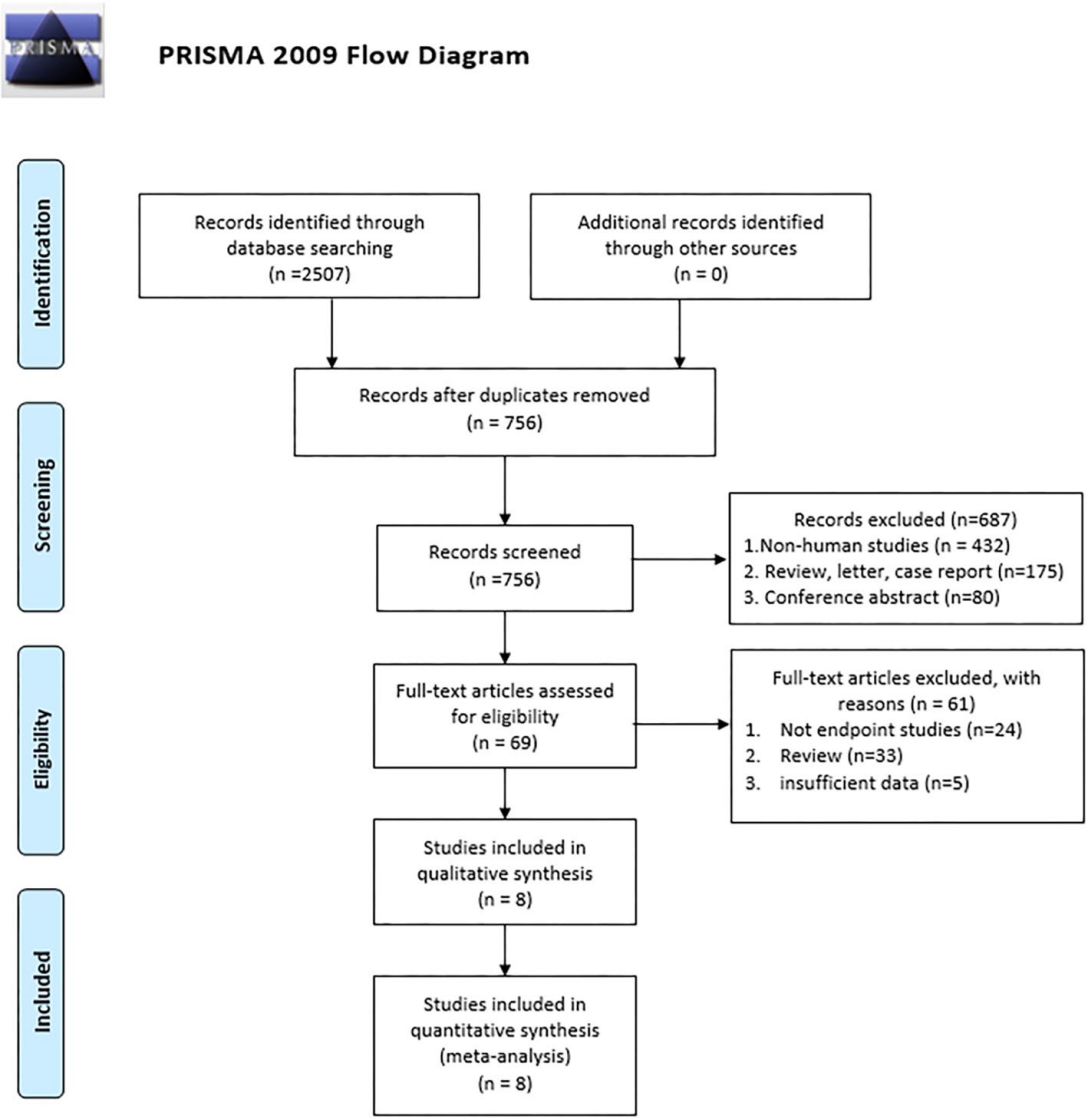
Flow chart of study selection process

We obtained 2507 articles by searching PubMed, Embase, Cochrane Library, Scopus, MEDLINE, and Web of Science, with 756 records saved after duplicates were removed. We then strictly filtered the articles by inclusion and exclusion criteria. We obtained 69 articles after excluding those that were not human studies, an inappropriate type of article and irrelevant articles by reading the title and abstract. By reading the full text of 69 articles, 24 articles were found to have no available outcome indicators, 33 reviews, and 5 papers with insufficient data. Eventually, a total of 1458 patients were included in the final eight studies [21, 27–33]. The study selection process is shown in Fig. 1.

### Characteristics of eligible studies

We have summarized the extracted data in Tables 1 and 2. The quality of all studies is listed in the table (Additional file 2: Table S1), and all studies were of a quality greater than 5. The included studies were all published between 2009 and 2019. None of the patients had received radiotherapy or chemotherapy, and all studies analyzed Slug protein expression using immunochemical (IHC) staining. The criteria used to assess Slug protein expression in these studies remain inconsistent, with four studies [10, 14, 22, 23] determining expression levels by the product of the percentage of positive cells scored and the intensity of staining score, three studies [24, 25, 27] determining Slug expression thresholds based only on the percentage of cells showing immunoreactivity, and one study [26] not describing the judging method in detail. Therefore, we cannot give a uniform threshold for high or low Slug protein expression, and we can only summarize the results of each study.

**Table 1:** Characteristics of studies included

Author	Year	Country	MA (Year)	Stage	NP	MF (month)	Cut-off	Dilution	Location	Positive rate	Survival endpoints	HR(e)	NOS
Liu [10]	2013	China	52	I–IV	441	NR	Scores > 4	NR	NR	39.50%	OS, PFS	Reported	8
Wan [14]	2017	China	NR	NR	314	240	Scores ≥ 4	NR	nuclear	75.90%	OS, PFS	Reported	9
Wu [22]	2019	China	55.3	I–III	137	NR	Scores ≥ 3	1:50	nuclear	24.10%	OS, PFS	Curve	8
Gu [23]	2019	China	NR	I–IV	108	88.5	Scores ≥ 4	1:600	cytoplasm	46.30%	OS, PFS	Curve	9
Prasad [24]	2009	India	56	NR	98	NR	> 10%	1:50	nuclear	34%	NR	NR	7
Cao [25]	2015	China	NR	I–IV	200	NR	> 10%	1:50	nuclear	42%	NR	NR	7
Ito [27]	2015	Japan	54	IV	47	61	> 5%	1:500	nuclear	40%	PFS	Reported	9
Wu [26]	2012	USA	NR	I–III	113	NR	NR	1:100	nuclear	44%	OS	Reported	6

*NR* not reported, *MA* mean age, *NP* No. of patients, *MF* median follow-up, *e* estimate, *Cut-off*: the threshold tumor cells was regarded as high expression, *Positive rate* proportion of high expression of slug protein, *HR(e)* the estimate of hazard ratio in the original articles, *NOS* the quality of the studies was assessed using the modified Newcastle Ottawa Scale (NOS)

**Table 2:** Data extracted from studies included

Study	Slug protein	Age (≤ 50/> 50)	Tumor size (≤ 2 cm/> 2 cm)	H grade (G1–G2/G3)	LN (P/N)	TNM stage (I–II/III–IV)	ER (P/N)	PR (P/N)	HER-2 (P/N)	HR
Liu 2013 [10]	High	45/33	45/33	21/57	57/21	43/35	NR	NR	NR	OS/DFS
	Low	30/25	39/6	30/15	15/30	37/8				
Wan 2017 [14]	High	55/77	71/61	90/42	62/70	NR	76/56	50/82	47/85	OS/DFS
	Low	19/23	21/21	34/8	11/31		26/16	10/ 32	19/23	
Wu 2019 [22]	High	17/16	12/21	24/9	17/16	NR	NR	NR	NR	OS/DFS
	Low	44/61	33/72	81/24	25/80					
Gu 2019 [23]	High	NR	40/18	44/14	21/37	47/11	44/14	29/29	17/40	OS/DFS
	Low		32/18	41/9	20/30	43/7	43/7	35/15	21/23	
Prasad 2009 [24]	High	22/11	NR	23/10	43/22	NR	NR	NR	NR	NR
	Low	40/25		33/32	19/14					
Cao 2015 [25]	High	NR	NR	43/29	39/33	57/15	31/41	34/38	52/20	NR
	Low			93/25	50/78	114/14	75/53	71/57	94/34	
Wu 2012 [26]	High	NR	NR	NR	NR	NR	NR	NR	NR	OS
	Low									
Ito 2015 [27]	High	8/15	5/18	NR	NR	NR	17/6	NR	NR	DFS
	Low	7/17	8/16				16/8			

The threshold of high or low expression of Slug protein was defined by each study. For specific methods, please refer to Table 1

*H* histological, *G* grade, *P* positive, *N* negative, *NR* no report, *LN* lymph nodes

### Correlation between slug expression and prognosis

**Fig. 2:**
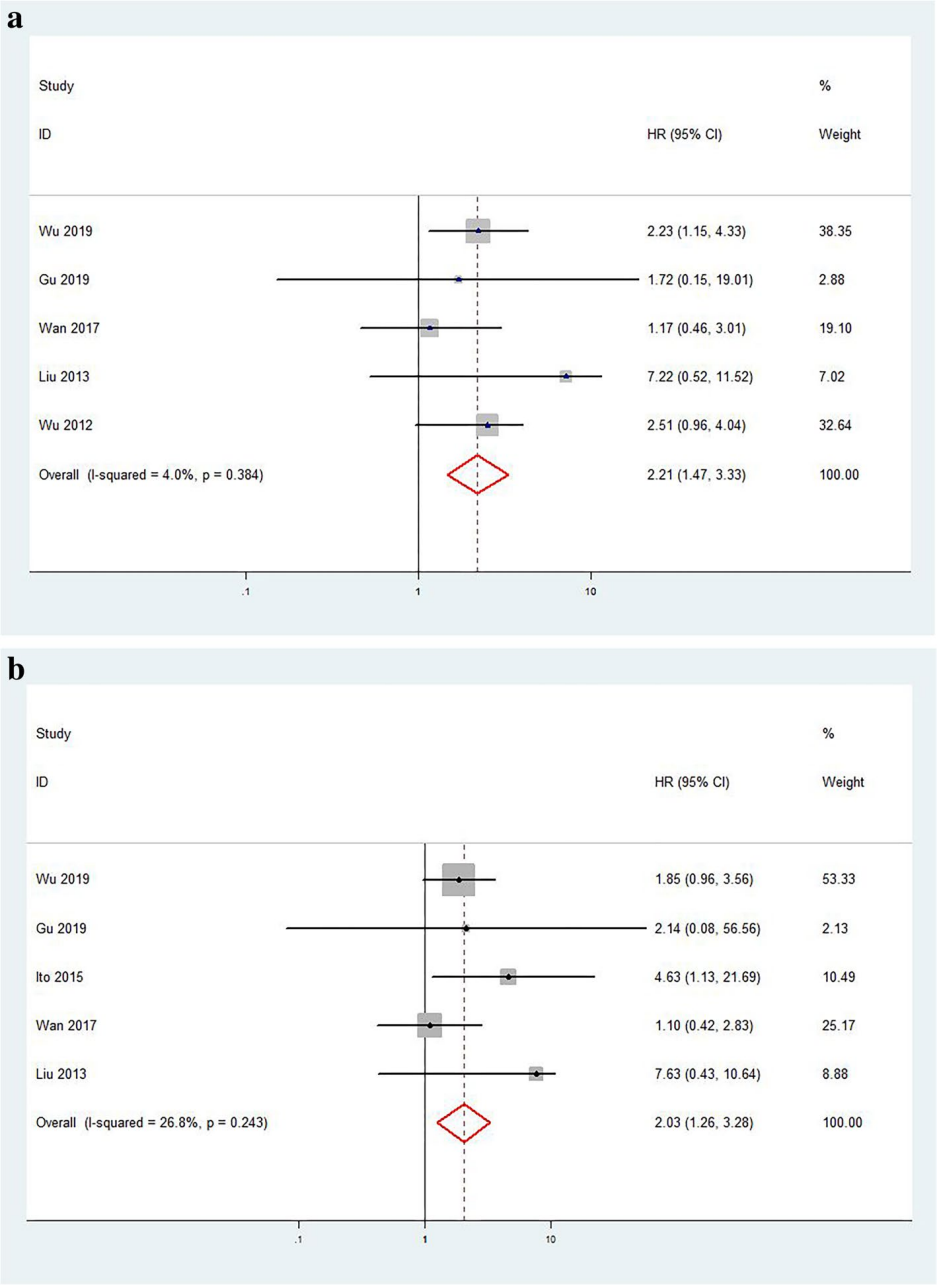
Forest plot depicting association between Slug protein expression and OS (**a**) and DFS (**b**) in breast cancer

A total of 5 articles provided OS-related data. Due to the low heterogeneity (*I*^2^ = 4.0%)，we used the fixed-effect model to pool HR, and the combined results showed that increased Slug protein expression was associated with poor OS (pooled HR = 2.21; 95% CI = 1.47–3.33; *P* < 0.001) (Fig. 2a). We did not perform subgroup analysis because of light heterogeneity (*I*^2^ < 50%). A total of five studies provided DFS-related data because there was low heterogeneity (*I*^2^ = 26.8%); we used the fixed-effect model to pool HR, and the combined results showed that elevated Slug protein expression was associated with poor DFS (pooled HR = 2.03; 95% CI = 1.26–3.28; *P* = 0.004) (Fig. 2b).

### Correlation between slug expression and clinicopathological parameters

A total of 7 articles provided related clinicopathological data; the specific characteristics are shown in Table 2. The clinicopathological parameter pooled OR values are shown in Table 3. The results showed that breast cancer patients with increased Slug protein expression had a higher TNM stage (I–II vs III–IV; pooled OR = 0.42; 95% CI = 0.25–0.70; *P* = 0.001) (Additional file 3: Figure S1a), were more prone to axillary lymph node metastasis (N+ vs N0; pooled OR = 2.16; 95% CI = 1.31–3.56; *P* = 0.003) (Additional file 3: Figure S1b) and had more severe ER deficiency (positive vs negative; pooled OR = 0.67; 95% CI = 0.45–0.99; *P* = 0.042) (Additional file 3: Figure S1c). However, this study shows that Slug protein expression is not associated with patient age (≤ 50 vs > 50; pooled OR = 1.15; 95% CI = 0.80–1.64; *P* = 0.455) (Additional file 4: Figure S2a), histological grade (I–II vs III; pooled OR = 0.58; 95% CI = 0.30–1.12; *P* = 0.104) (Additional file 4: Figure S2b), tumor size (≤ 2 cm vs > 2 cm; pooled OR = 0.91; 95% CI = 0.64–1.28; *P* = 0.577) (Additional file 4: Figure S2c), PR status (positive vs negative; pooled OR = 0.84; 95% CI = 0.38–1.85; *P* = 0.661) (Additional file 4: Figure S2d), and HER-2 status (positive vs negative; pooled OR = 0.70; 95% CI = 0.47–1.06; *P* = 0.089) (Additional file 4: Figure S2e).

**Table 3:** Relationship of Slug expression and clinicopathological parameters of breast cancer

Features	OR (95% CI)	*P*	*I*^*2*^	Model
Age (≤ 50 vs> 50)	1.15 (0.80, 1.64)	0.455	0.00%	Fixed
Histological grade (G1 + G2 vs G3)	0.58 (0.30, 1.12)	0.104	73.20%	Random
Tumor size (≤ 2 cm vs> 2 cm)	0.91 (0.64, 1.28)	0.577	63.80%	Random
LN (N+ vs N0)	2.16 (1.31, 1.56)	0.003	61.80%	Random
TNM (I–II vs III–IV)	0.42 (0.25, 0.70)	0.001	0.60%	Fixed
ER status (positive vs negative)	0.67 (0.45, 0.99)	0.042	0.00%	Fixed
PR status (positive vs negative)	0.84 (0.38, 1.85)	0.661	72.90%	Random
HER-2 status (positive vs negative)	0.70 (0.47, 1.06)	0.089	0.00%	Fixed

### Sensitivity analysis and publication bias

**Fig. 3:**
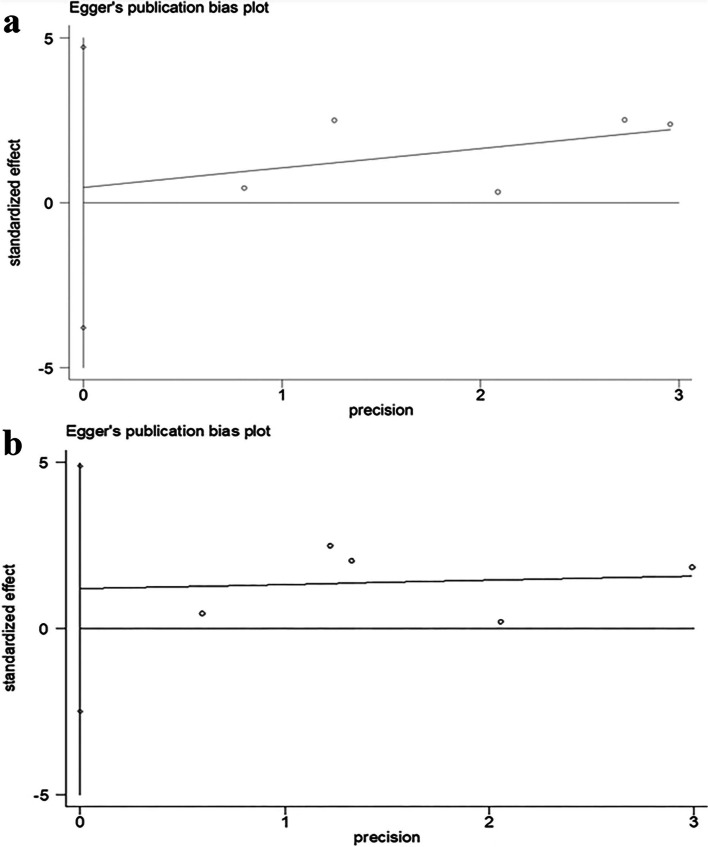
Funnel plot of Egger’s test for publication bias: OS (**a**) and DFS (**b**)

Our analysis of publication bias using Egger’s test correlation test revealed no bias for OS (*P* = 0.751) (Fig. 3a) and DFS (*P* = 0.596) (Fig. 3b). The sensitivity analysis showed that the results were reliable for OS (Additional file 5: Figure S3a) and DFS (Additional file 5: Figure S3b).

## Discussion

We used this meta-analysis to determine the relationship between slug expression in breast cancer and prognostic and clinicopathological parameters. Here, we selected eight articles involving 1458 patients. Our meta-analysis showed that increased Slug protein expression in breast cancer was associated with poorer OS (combined HR = 2.21; 95% CI = 1.47–3.33; *P* < 0.001) and DFS (combined HR = 2.03; 95% CI = 1.26–3.28; *P* = 0.004). To further explore the role of Slug protein in breast cancer, we further analyzed the relationship between its expression and clinicopathological parameters of breast cancer. The results showed that patients with increased Slug expression in breast cancer tended to have a higher TNM stage (I–II vs III–IV; combined OR = 0.42; 95% CI = 0.25–0.70; *P* = 0.001) and were more prone to axillary lymph node metastasis (N+ vs N0; combined OR = 2.16; 95% CI = 1.31–3.56; *P* = 0.003). Therefore, we hypothesize that a high level of Slug protein expression induces tumor metastasis and progression via various pathways, thus leading to poor cancer patient prognosis. Hence, Slug expression may be a biomarker for breast cancer patient prognosis. In addition, there was no significant heterogeneity in our primary outcomes OS and DFS. Sensitivity analysis suggests that our study is stable and credible.

Slug has been associated with the prognosis of a variety of cancer cells. Liu et al. [34] and Song et al. [35] found that Slug expression was higher in lung cancer cells than in normal lung tissue and that increased expression of Slug in lung tumor cells was associated with poorer survival and more aggressive clinicopathological parameters. Chang et al. [36] and Gu et al. [37] found that slug is highly correlated with the invasiveness and drug resistance of ovarian cancer cells. Toiyamo et al. [38] found that Slug protein expression was significantly elevated in colorectal cancers with high T-stage, liver metastases, and lymph node metastases and may be a potential prognostic marker for colorectal cancer. A previous study analyzed the relationship between Slug expression and solid tumors [39] and found that Slug was associated with poor prognosis in lung, head, neck, urological and gastrointestinal cancers, but not breast cancers. Due to the early stage of the study, only two studies related to Slug were included, and we had eight studies with strict inclusion criteria; our study is more reliable.

Metastasis of tumor cells and resistance to anti-tumor therapy are the leading causes of poor prognosis in tumor patients. Ramaswamy et al. [40], Pan et al. [13], and Shao et al. [41] demonstrated that Slug is an essential factor in promoting breast cancer cell metastasis and may be an important marker of metastatic potential. Slug was initially recognized as a member of the EMT because of its involvement in the EMT project. During breast cancer progression, cells and cell adhesion are lost in the EMT process, leading to migration and invasion [20, 42]. There are many molecular mechanisms by which slug promotes the metastasis of cancer underlying EMT. Liu et al. [43] found that slug inhibited the expression of miR-200b and miR-1, and that inhibition of miR-200b and miR-1 promoted EMT and tumor cell invasion. Fazilaty et al. [16] found that slug could induce TNC through a signaling cascade, thereby promoting tumor cell invasiveness. Moreover, Lamouille et al. [44] found that high expression of slug reduced the expression of epithelial genes and activated the expression of mesenchymal genes, thus promoting tumor cell metastasis.

High slug expression can lead to multiple drug resistance [17, 18]. Slug has recently been found to play an important role in tamoxifen resistance to breast cancer [45, 46]. Slug expression prevented tamoxifen's killing effect on ER (+) breast cancer cells [47]. Slug has been shown to induce endocrine therapy resistance in breast cancer cells by altering cell survival signaling pathways, leading to worse DFS [48]. Musgrove et al. found that the loss of ER expression due to increased slug expression is the leading cause of drug resistance to tamoxifen [49]. Some studies suggested that slug induces tamoxifen resistance by increasing EGFR expression and Erk phosphorylation [50]. Moreover, Li et al. [51] found that slug can induce chemotherapy resistance in cancer cells via the PI3K/Akt/GSK3b pathway. These studies confirm that slug leads to poor prognosis in breast cancer, consistent with our meta-analysis results.

Our meta-analysis shows that Slug is a crucial biomarker for predicting prognosis in breast cancer patients, which is the main finding of this study. No heterogeneity or publication bias was found in this meta-analysis, and sensitivity analysis suggested that our results were reliable. There were limitations in our meta-analysis. First, the cut-off values of low and high expression of Slug were diverse among those studies. Hence, more large-scale, well-designed studies are warranted to confirm our results. Second, there were differences in the type and dilution of immunohistochemical antibodies. Third, the HR reliability of some prognostic parameters obtained from the Kaplan-Meier curve was poor. Fourth, even though we screened through 1186 articles, we ended up with only eight available studies, and only one of them from a non-Asian population, so more studies are needed in non-Asian populations in the future. Fifth, although we searched as far as possible for available studies, unfortunately, only eight studies met the inclusion requirements. This has led to our results being inconclusive, but there is no doubt that our results provide some insight into future research.

## Conclusion

Our meta-analysis identified for the first time that increased Slug expression may predict poor survival and is associated with advanced TNM stage, lymph node metastasis, and more severe ER deficiency in patients with breast cancer. Therefore, we have reason to believe that Slug is not only an indicator of patient prognosis but may also be a new target for breast cancer therapy. Regarding the shortcomings of our meta-analysis, we expect further studies with larger sample sizes to verify our results.

## Supplementary Information

**Additional file 1: Supplementary file.** The general method of medical studies meta-analysis [1]

**Additional file 2: Table S1.** Quality assessment of the included studies.

**Additional file 3: Figure S1.** Forest plot showed the association between Slug protein expression and TNM stage (a), LN status(b), ER status(c) in breast cancer.

**Additional file 4: Figure S2.** Forest plot depicting association between slug protein expression and Age (a), Histological grade(b), Tumor size(c), PR status(d), HER-2 status(e) in breast cancer.

**Additional file 5: Figure S3.** Sensitivity analysis of meta-analysis of the association of Slug protein expression with OS (a) and DFS (b) in breast cancer patients.

## Data Availability

The datasets used and/or analyzed during the current study are available from the corresponding author on reasonable request.

## Abbreviations

OS: Overall survival
DFS: Disease-free survival
HR: Hazard ratio
OR: Odds ratio
CI: Confidence interval
LN: Lymph nodes
ER: Estrogen receptor
PR: Progesterone receptor
HER-2: Human epidermal growth factor receptor 2
