# Supplementary material for: Prognostic and clinicopathological value of Slug protein expression in breast cancer: a systematic review and meta-analysis
Source: World J Surg Oncol. 2022 Nov 14;20:361. doi: 10.1186/s12957-022-02825-6 (PMC9661812; doi:10.1186/s12957-022-02825-6)
Supplement: Supplementary file 1 — Additional file 1: Supplementary file. The general method of medical studies meta-analysis [1] [file 12957_2022_2825_MOESM1_ESM.doc]

**A detailed explanation of the meta-analysis**

Meta-analysis (Meta-analysis) is a statistical method used to compare and synthesize the results of studies on the same scientific question, and the significance of the conclusions depends on the quality of the included studies. By combining all relevant studies, the effects of health care can be estimated more accurately than in individual studies, and it is helpful to explore the consistency of the evidence across studies and the differences between studies.

The meta-analysis includes (i) analysis of heterogeneity and tests of statistical consistency in handling multiple independent studies; (ii) calculation of combined effect values; (iii) tests of combined effect values.

The test of heterogeneity is an essential part of Meta-analysis. Q-test is mainly used to determine whether the heterogeneity of multiple independent studies is statistically significant, and it is generally considered that when P>0.1, the results of each independent research are in good agreement. The selection of effect values is usually determined according to the nature of the clinical study and the type of data, and there are two types of statistical models commonly used for combining effect values in Meta-analysis: fixed-effects models and random-effects models. If the heterogeneity could not be resolved after the analysis and treatment, a random-effects model could be adopted to calculate the combined effect value.

Hypothesis testing is carried out to test whether the combined effect of multiple similar studies is statistically significant. Commonly used methods are as follows: (i) z(u) test: if P≤0.05, the combined statistic of multiple reflections is statistically substantial; (ii) confidence interval method: when the test effect indicator is OR, RR, its 95% confidence interval is equivalent to P<0.05 if it does not contain 1, which means it is statistically significant. When the testing effect indicators are RD and SMD, the 95% confidence interval is equivalent to P<0.05 if it does not contain 0, statistically significant.

Meta-analyses, especially those synthesizing high-quality randomized controlled trials (RCTs), are considered high-level evidence for evidence-based medicine and have the following functions: (i) to achieve quantitative synthesis; (ii) to provide a systematic and reproducible synthesis of the same question; (iii) to improve the statistical validity of the initial results by synthesizing the results of multiple small sample studies on the same topic; (iv) to address inconsistencies in study results and improve effect estimates; (v) to answer questions not asked by the original studies; (vi) to explore the extent of publication bias in the existing literature; and (vii) to raise new research questions and provide direction for further research.

However, meta-analysis has some limitations. Meta-analysis may be meaningless when the original study is of poor quality, and the combined results may be questioned as "rubbish in, rubbish out." The quality of the primary literature is the key to a systematic review. Poor quality clinical evidence should be recognized as a limitation, treated cautiously, and targeted with high-quality clinical studies to improve and enrich the evidence base. When clinical differences exist across original studies, it does not make sense to combine all included studies in a single Meta-analysis, such as when conducting mixed comparisons between different treatment measures and control measures requiring consideration of the combination of each comparison, when the decision to combine cannot be dependent on statistical methods but requires discussion and clinical judgment.

**The general method of medical studies meta-analysis[1]**

| 1. Screening-related studies: PubMed, EMBASE, Web of Science, and Scopus, *et al.* |
| --- |
| 1. Heterogeneity of studies: Cochran Q test, *I*^2^ |
| 1. Meta-analysis: fixed/random effect model, Forrest plot |
| 1. Evaluate sources of the heterogeneity: subgroup analysis, sensitivity test, meta-regression |
| 1. Check publication bias: funnel plot, Egger’s regression test, trim and fill method |
| 1. Present meta-analysis result based on PRISMA |

Reference

1. Lee YH. An overview of meta-analysis for clinicians. The Korean journal of internal medicine. 2018;33(2):277-83. Epub 2017/12/27. doi: 10.3904/kjim.2016.195. PubMed PMID: 29277096; PubMed Central PMCID: PMCPMC5840596.
