# Supplementary material for: Prognostic and clinicopathological value of Slug protein expression in breast cancer: a systematic review and meta-analysis
Source: World J Surg Oncol. 2022 Nov 14;20:361. doi: 10.1186/s12957-022-02825-6 (PMC9661812; doi:10.1186/s12957-022-02825-6)
Supplement: Supplementary file 2 — Additional file 2: Table S1. Quality assessment of the included studies. [file 12957_2022_2825_MOESM2_ESM.doc]

**Table S1. Quality assessment of the included studies.**

| Study | Selection | | | | Comparability | Exposure | | | Scores |
| --- | --- | --- | --- | --- | --- | --- | --- | --- | --- |
|  | A | B | C | D | E | F | G | H |  |
| Liu 2013^[10]^ | * | * | * | * | * | * | * | * | 8 |
| Wan 2017^[14]^ | * | * | * | * | ** | * | * | * | 9 |
| Wu 2019^[22]^ | * | * | * |  | ** | * | * | * | 8 |
| Gu 2019^[23]^ | * | * | * | * | ** | * | * | * | 9 |
| Prasad 2009^[24]^ | * | * | * | * | ** | * |  |  | 7 |
| Cao 2015^[25]^ | * | * | * | * | ** | * |  |  | 7 |
| Wu 2012^[26]^ |  | * | * | * | * | * |  |  | 6 |
| Ito 2015^[27]^ | * | * | * | * | ** | * | * | * | 9 |

| Note: "*" is a score. In the selection, a represents the representative of the exposed queue (consecutive patients)(*), B represents the selection of the non-exposed queue(*), C represents  the determination of exposure(*), D represents that no outcome event occurred to the study object before the study began(*), E indicated the study controlled for significant confounders or other confounders(**), F indicated the assessment of outcome events, G indicated the adequacy of  follow-up (median follow-up＞ 5years)(*), H indicated the completeness of follow-up(＜5%)(*). |  |
| --- | --- |
|  |  |
|  |  |
|  |  |
|  |  |
|  |  |
|  |  |
|  |  |
