# Supplementary figures and images for: Prognostic and clinicopathological value of Slug protein expression in breast cancer: a systematic review and meta-analysis
Source: World J Surg Oncol. 2022 Nov 14;20:361. doi: 10.1186/s12957-022-02825-6 (PMC9661812; doi:10.1186/s12957-022-02825-6)

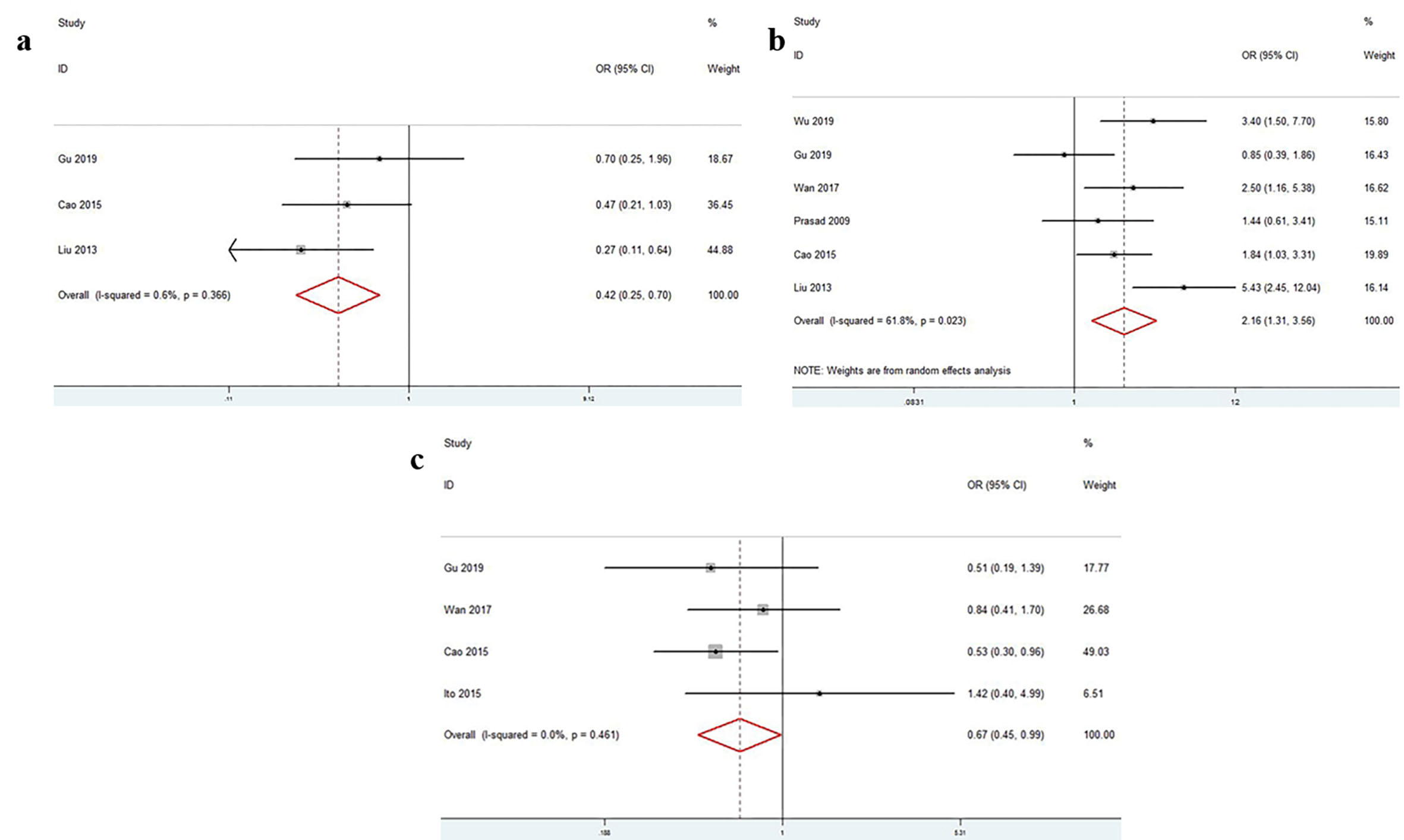

Supplement: Supplementary file 3 — Additional file 3: Figure S1. Forest plot showed the association between Slug protein expression and TNM stage (a), LN status(b), ER status(c) in breast cancer. [file 12957_2022_2825_MOESM3_ESM.jpg]

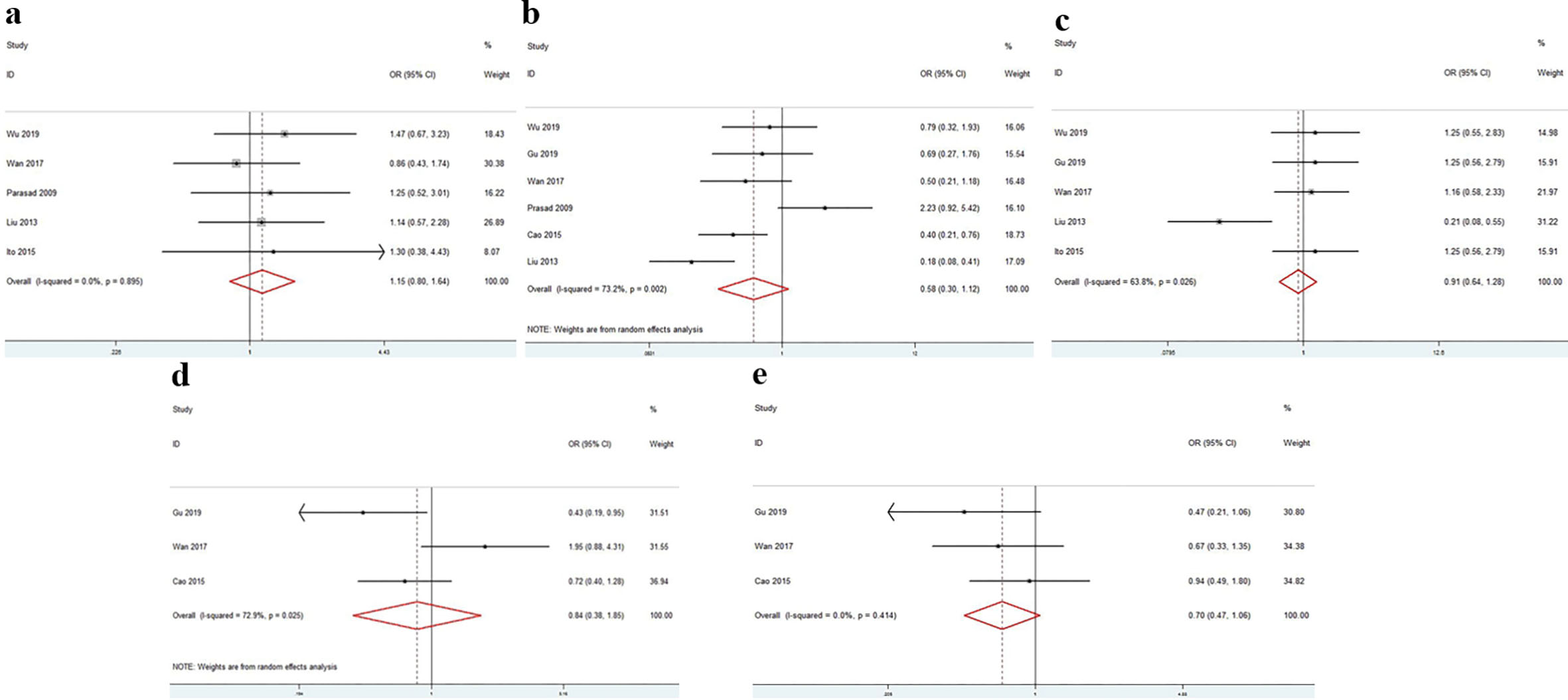

Supplement: Supplementary file 4 — Additional file 4: Figure S2. Forest plot depicting association between slug protein expression and Age (a), Histological grade(b), Tumor size(c), PR status(d), HER-2 status(e) in breast cancer. [file 12957_2022_2825_MOESM4_ESM.jpg]

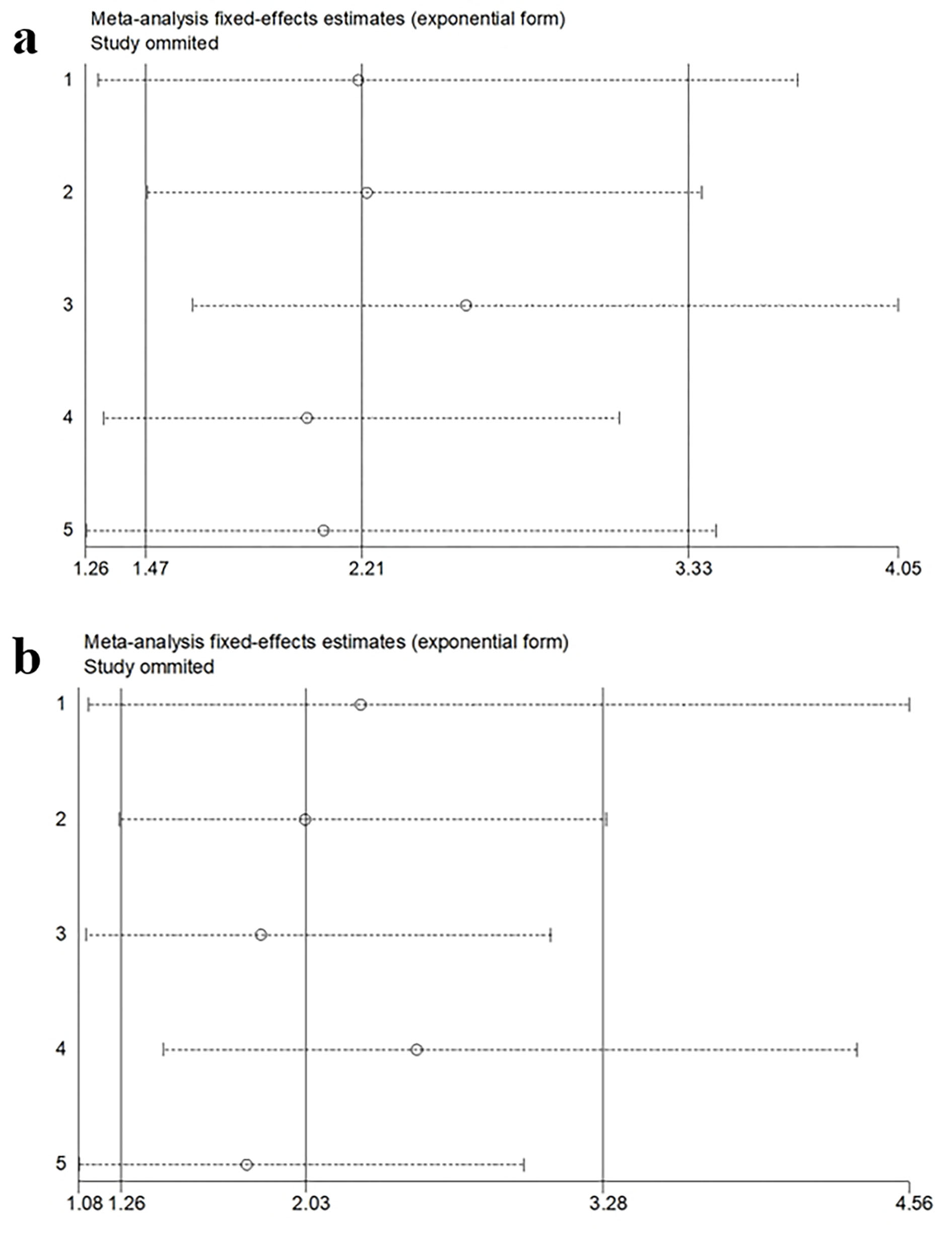

Supplement: Supplementary file 5 — Additional file 5: Figure S3. Sensitivity analysis of meta-analysis of the association of Slug protein expression with OS (a) and DFS (b) in breast cancer patients. [file 12957_2022_2825_MOESM5_ESM.jpg]
